# Trends and causes of neonatal mortality from 2010 to 2017 at a Health and Demographic Surveillance site in Northern Ethiopia

**DOI:** 10.1080/16549716.2023.2289710

**Published:** 2023-12-21

**Authors:** Mengistu Welday Gebremichael, Mache Tsadik, Haftom Temesgen Abebe, Abraha Gebreegzabiher, Selam Beyene, Abera Berhe Aregawi, Solomon Weldemariam

**Affiliations:** College of Health Sciences, Mekelle University, Mekelle City, Tigray, Ethiopia

**Keywords:** Trends, causes of death, neonatal mortality, Tigray, first week of life

## Abstract

**Background:**

Half of global under-five mortalities is neonatal. The highest rates are found in low-income countries such as Ethiopia. Ethiopia has made progress in reducing under-five mortality, but neonatal mortality remains high. Evidence collected continuously at the community level is crucial for understanding the trends and causes of neonatal mortality.

**Objectives:**

To analyse the trends and causes of neonatal mortality at the Kilte-Awlelo Health and Demographic Surveillance System (KAHDSS) site in Ethiopia from 2010 to 2017.

**Methods:**

A descriptive study was conducted using data from neonates born between 2010 and 2017 at the KAHDSS site. Data were collected using interviewer-administered questionnaires. Causes of death were examined, and neonatal mortality trends were described using simple linear regression.

**Results:**

The overall average neonatal mortality rate was 17/1000 live births (LBs). The rate increased from 12 per 1000 LBs in 2010 to 15 per 1000 LBs in 2017. The majority of neonatal deaths occurred during the first week of life, and more than one-half died at home. The leading causes were sepsis, pre-term birth (including respiratory distress), disease related to the perinatal period, birth asphyxia, and neonatal pneumonia.

**Conclusions:**

The high neonatal mortality in Ethiopia requires urgent attention and action. Sepsis, preterm birth, perinatal diseases, asphyxia, and neonatal pneumonia are the leading causes of death in neonates. Facility- and community-based health services should target the leading causes of neonatal deaths.

## Background

The neonatal period, defined as the first month (28 days) of life, is the most critical period for survival. Globally, in 2017, there were 2.5 million neonatal deaths, most of which occurred early in the first days of life. Neonatal mortality contributed to 47% of under-five mortality. The global neonatal mortality rate (NMR) had declined from 37 per 1000 live births (LBs) in 1990 to 18 per 1000 LBs in 2017 [1]. Although this overall reduction in mortality is over 50%, this drop is slower than that in children older than one month [2]. The highest rates are in sub-Saharan Africa (SSA) and South Asia [1].

Over the past decade, Ethiopia has improved many health indicators due to well-coordinated and extensive efforts made by the government, community, and partners through the health extension programmme and the expansion of primary health care. As a result, Ethiopia has achieved Millennium Development Goal-4 by reducing under-five mortality by two-thirds three years before the 2015 deadline [3]. Although neonatal mortality has declined;, it remains high and Ethiopia is among the five countries where half of the world’s neonatal deaths are concentrated [1]. The 2016 Ethiopian Demographic Health Survey (EDHS 2016) reported the national NMR at 29 per 1000 LBs, this being a fall of about 25% since 2005. Over the same time period, under-five and infant mortality rates declined by about 45% and 38%, respectively [4,5]. The EDHS 2011 report showed significant regional variation [6] and Tigray’s was higher, and the finding was substantiated by the local survey report that was 49.2 [7]. Analysis of birth history information for LBs from the 2000, 2005, and 2011 EDHS reports showed that the NMR declined by 1.9% per annum from 1995 to 2010, while the early NMR declined by 0.9% per annum [8]. A comparison between the most recent EDHS in 2019 and the 2016 EDHS reveals an increase of 3.5% in neonatal mortality. In contrast, under-five and infant mortality rates have declined at a similar rate as seen in earlier EDHS reports [9].

The EDHS, clinical-based reports, and small-scale survey studies, which require attention and triangulation, are the most commonly used sources of information for neonatal mortality in Ethiopia [10]. However, many demographic and health-related events occur at the community level [11]. The Health and Demographic Surveillance System (HDSS) generates vital community-level data on births, deaths, migration, and health service utilisation. This information is used by planners and policy-makers in developing evidence-based interventions in resource-limited settings [12].

The three major causes of neonatal deaths worldwide are infections (36%), pre-term delivery (28%), and birth asphyxia (23%), with some variation between countries [13]. In a study conducted in Nepal, Bangladesh, Malawi, and India, the major cause of neonatal death in Nepal and Bangladesh was infections, whereas it was prematurity in Malawi and birth asphyxia in India [14]. Similarly, a prospective cohort study conducted in selected hospitals in Northern Ethiopia found that the major causes of neonatal mortality were prematurity (34%), asphyxia (31%), and infections (12%) [15].

Various predictors of neonatal mortality have been assessed in different countries. Analysis of Nepal’s Demographic and Health Survey from 2001 to 2011 found maternal education, birth interval, number of antenatal care (ANC) visits, maternal height, and exposure to indoor air pollution to be significant determinants of neonatal mortality [16]. Studies conducted in Ethiopia have found that increased neonatal mortality is associated with multiple births, not attending ANC visits, neonates born by caesarean section, and not initiating breastfeeding within one hour of birth [17]. A study analysing the determinants of neonatal mortality based on the EDHS in 2000, 2005, and 2011 found that male sex, maternal age, birth interval, number of tetanus toxoid vaccinations, and maternal education were independent predictors of neonatal mortality [8].

There is a need to collect data continuously in order to better understand the complexities and trends. A case in point is this study that used the Kilte-Awlalo Health and Demographic Surveillance System (KAHDSS). KAHDSS was established in 2009 by Mekelle University to generate evidence on key health indicators including neonatal mortality. The purpose of this paper is to generate reliable information for policy- and decision-makers to assist efforts aimed at reducing the neonatal mortality. The aim is to analyse the trends and causes of Neonatal Mortality at the KAHDSS site in Ethiopia from 2010 to 2017.

## Methods

### Study design and setting

A descriptive study using longitudinal epidemiologic data from KAHDSS was conducted on neonates born between 2010 and 2017 in the Tigray region, North Ethiopia. KAHDSS site consists of three districts (Klite-Awulaelo, Atsebi, and Wukro) having 12 kebelles. In 2017, there were 78,579 residents in the site. During the establishment in 2009, there were ten kebelles having 14,453 households with a total population of 66,453.

### Data collection

Data were collected using interviewer-administered, pre-tested, structured, and standardised questionnaires administered by 35 trained full-time field workers (high school completed, trained, and residents of the districts) and directed by four field supervisors who scrutinised the data quality. Causes of death were categorised according to the International Classification of Diseases (ICD-10) by two paediatricians. Whenever there was a discrepancy, a third senior paediatrician confirmed the diagnosis. Interviews were administered to the heads of the households (father or mother) or the next oldest family member. House-to-house visits were made in order to capture information regarding neonatal mortality. Each household was visited every month to collect data on events, and this information was updated every six months. When an event occurred, the relevant information was usually collected within 30–40 days of the event with due consideration and sensitivity for the period of mourning. Neonatal mortality was estimated using information on the birth histories based on the child’s date of birth, survivor status, and age at death for the deceased children.

There were 11,306 live-born infants during the eight-year study period. A live birth is so defined when a foetus exits the mother, showing any definite signs of life, such as voluntary movement, heartbeat, or pulsation of the umbilical cord. This can be for a brief time period regardless of whether the umbilical cord or placenta is intact. The NMR is the number of neonatal deaths (within the first 28 days of life) per 1000 LBs.

### Statistical analysis

HRS2 software was used for data entry, and STATA version 14.1 was used for cleaning and analysis. A descriptive analysis of trends and causes of death was conducted using simple linear regression, frequencies, percentages, and graphs. The NMR trend was examined using Locally Weighted Scatterplot Smoothing (LOWESS Smoothing) by creating a smooth line through the time plot.

## Results

### Trends of neonatal mortality

**Figure 1.**
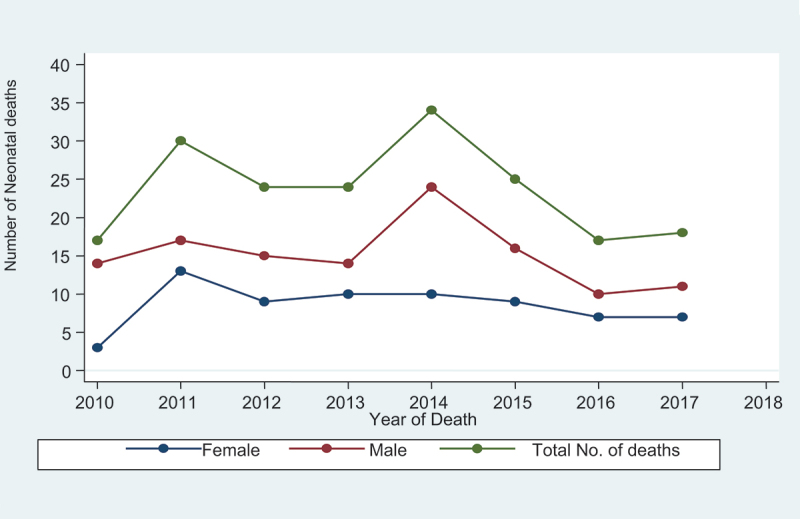
Trends in neonatal mortality by sex from 2010 to 2017 in KAHDSS Tigray, Ethiopia.

Of the 11,306 LBs, 189 neonatal deaths (17 per 1000 LBs) were in the study area during the eight consecutive years (2010–2017) under investigation (Table 1). The number of mortalities among males was higher than among females (Figure 1). Of the neonatal deaths, 63.7% (123) were males, and 96.9% (187) resided in the rural areas of the surveillance site. More than one-half, 56.9% (110), died at home, 41.9% (81) died in health facilities, and the remaining 1.2% (2) died in other locations.

**Table 1.** Neonatal mortality rates from 2010 to 2017 in KAHDSS Tigray, Ethiopia.

Year	Deaths	Early neonatal deaths	Late neonatal deaths	Neonatal deaths among females	Neonatal deaths among males	Number of live births	Total NMR*	LOWESS smoothed NMR
2010	17	14	3	3	14	1,417	12	12.0
2011	30	24	6	13	17	1,516	20	16.6
2012	24	18	6	9	15	1,520	16	17.4
2013	24	19	5	10	14	1,437	17	18.4
2014	34	28	6	10	24	1,457	23	19.6
2015	25	18	7	9	16	1,465	17	17.6
2016	17	13	4	7	10	1,286	13	14.7
2017	18	15	3	7	11	1,208	15	15.0
2010–2017	189	149	40	68	121	11,306	17	

*NMR: Neonatal mortality rate.

**Figure 2.**
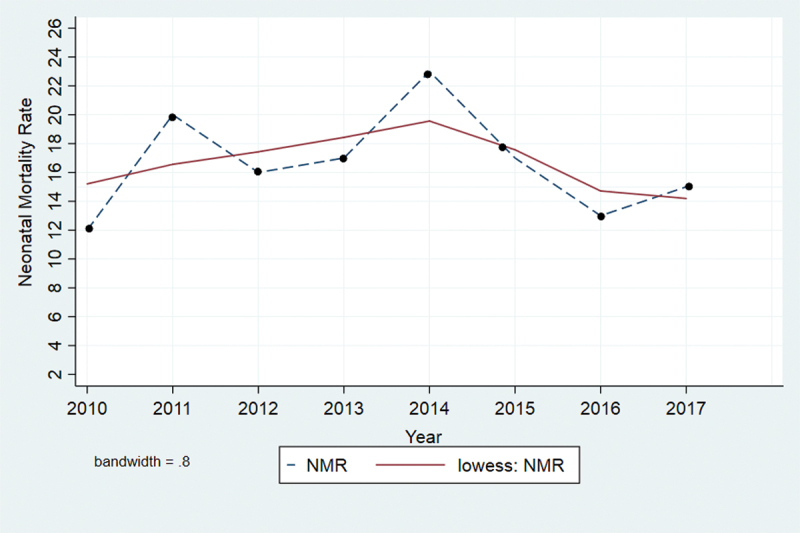
Trend in neonatal mortality rate in Kilite-Awlaelo HDSS site in Northern Ethiopia, 2010 and 2017.

The mean NMR for the eight-year study period was 16.63 (Std. Dev + 3.58) and in the range of 12–23. The NMR had increased from 12 per 1000 LBs in 2010 to 23 per 1000 LBs in 2014, which showed a 92% increase, followed by a decline of 35% from 2014 to 2017. The overall NMR had increased by 25% from 12 per 1000 LBs in 2010 to 15 per 1000 LBs in 2017 (Table 1 and Figure 2). However, the simple linear regression analysis shows that the increase was not statistically significant (*p*-value = 0.924).

**Figure 3.**
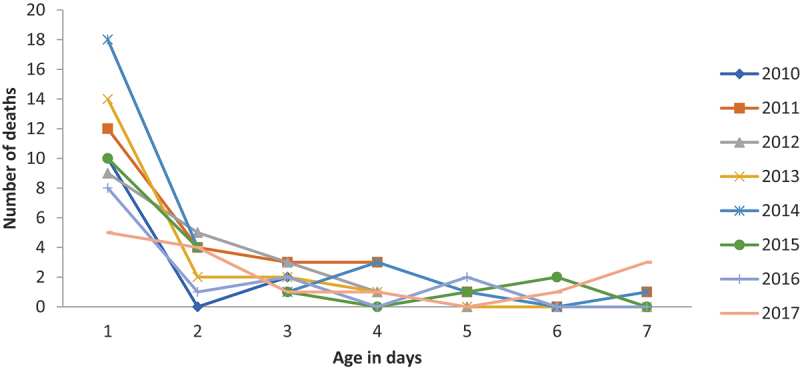
Age pattern of early neonatal deaths from 2010 to 2017 in KAHDSS Tigray, Ethiopia.

Early neonatal deaths, occurring during the first seven days of life, accounted for 79% of all neonatal deaths, suggesting that the slight falling trend noted for overall neonatal mortality is possibly attributable to the decline in the early neonatal period (Figure 3).

### Causes of neonatal mortality

**Figure 4.**
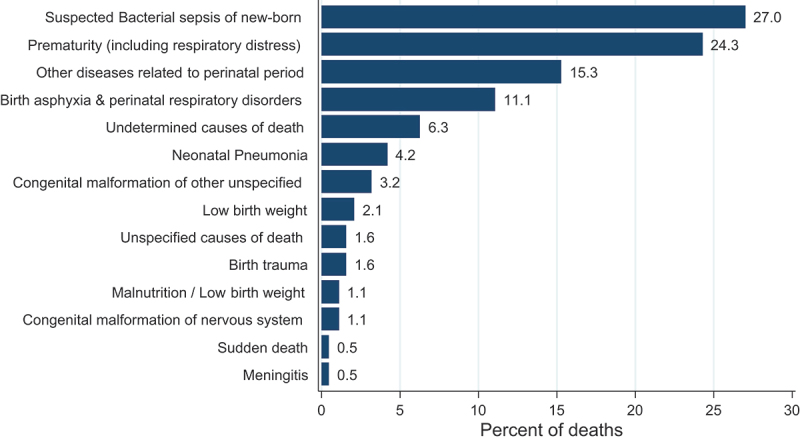
Causes of neonatal mortality from 2010 to 2017 in KAHDSS Tigray, Ethiopia.

The study indicated that the leading causes of death in the study area from 2010 to 2017 were suspected bacterial sepsis of the newborn (27.0%), prematurity including respiratory distress (24.3%), diseases related to the perinatal period (15.3%), birth asphyxia and perinatal respiratory disorders (11.1%), and neonatal pneumonia (4.2%) (Figure 4).

## Discussion

The analysis revealed that the average NMR in the study area was 17/1000 LBs over eight consecutive years. Male mortality was prevalent compared to females, and almost all deaths occurred in rural settings. A large percentage of early neonatal mortality was observed. NMR has increased by 25% from 2010 to 2017. In this study, the leading causes of death were sepsis, preterm birth (PTB), including respiratory distress, disease related to the perinatal period, birth asphyxia and perinatal respiratory disorders, and neonatal pneumonia.

The cumulative average of NMR over the study period was lower when compared to similar studies conducted in Asia and Africa. For instance, NMR was reported in Bangladesh (32.3/1,000 LBs), in Nigeria (39/1000 and 38/1000 LBs), and in Kenya (28.5/1000 LBs) [18–21]. HDSS reports from different parts of Ethiopia also show a high rate of NM. It was reported as 28/1000 LBs in Eastern Ethiopia, 35.5/1000LBs in Jima, 24.8/1000LB nationally, and 23.2/1000LBs in the global burden of diseases study [22–25]. It is also lower than EDHS findings from Ethiopia, where 29/1000 LBs in 2016 and 30 deaths per 1,000 LBs in the mini EDHS 2019 [4,9]. Observed NM discrepancies could be attributed to the timing and setting differences between studies. On the other hand, the lower rate of NM of this study could be partly justified because the present study was conducted in the HDSS site, which is located near Mekelle, the capital city of the Tigray region, and has a general hospital. Therefore, the population living in such a setting could have better access to health care, quality of care, and better awareness about their health.

However, our findings are in line with those of the 2017 UN Inter-agency Group, and Hug’s study in 2019. Both studies reported NMRs of 18/1000 LBs [1,24] with higher rates in SSA and South Asia. Even though progress has been made on NMR reduction in the past three decades, accelerated improvements are still essential to achieve the SDG target by reducing the NMR to 12/1000 LBs by 2030 [26].

Even though the cumulative average rate of neonatal mortality decreased throughout the study period compared to most national and regional studies, the trend of NMR increased by 25% from 2010 to 2017. This could be partly due to high fertility and population growth. The World Bank report revealed that SSA’s share of global child mortality is expected to increase by 2030 due to a high number of births in the region [27].

The number of deaths was higher among males than females. This finding aligns with the previous studies conducted in Asia, Africa, and Ethiopia [4,14,17,21,22,25]. Biological factors are one possible explanation. A study conducted in Taiwan reported that the more significant risks of PTB, operative delivery, neonatal death, macrosomia, and congenital anomalies were common among male neonates [28]. Another study also revealed that respiratory morbidity and mortality due to respiratory problems are more common among preterm males compared to females [29].

Early neonatal deaths accounted for a large percentage of all deaths; most occurred in rural settings and were at home. The findings are congruent with reports from the UN Inter-agency Group, where nearly three-quarters of all neonatal deaths in 2017 occurred in the first week of life [1]. Reports from African and Asian countries and different parts of Ethiopia also confirm these findings [8,14,17–19,21–23,25]. This could be explained by the fact that most neonatal deaths are associated with maternal complications surrounding the perinatal period. Moreover, in Ethiopia, most deliveries take place at home [4] without the help of a skilled birth attendant. Therefore, this condition could intensify the NMR in the absence of neonatal care and neonatal intensive care unit (NICU) services.

In addition, this study assesses the leading causes of neonatal death. Reports from Ethiopia revealed that bacterial sepsis was the major cause of neonatal deaths [22], consistent with the current findings. Similarly, a cohort study conducted in the KAHDSS site from 2009 to 2013 also reported that bacterial sepsis, PTB, and birth asphyxia were the leading causes of neonatal deaths over four years of the study period [30]. However, the WHO 2019 report, including three other studies, revealed that PTB followed by birth asphyxia and infection were the leading causes of NM [14,15,31]. Congenital abnormality, trauma, and pneumonia are also listed as causes of mortality in these studies.

On the other hand, studies done in Ethiopia and Bangladesh reported that birth asphyxia was the leading cause of mortality, followed by PTB, sepsis, respiratory distress syndrome, and pneumonia [19,23,32]. Variation in the sequence of the reasons was observed in some literature. This variation could be because of differences in the configuration of services between countries and the availability of infrastructure and proximities to nearby referral health facilities. These findings imply that most deaths occurred due to inadequate quality of care during pregnancy, delivery, and postnatal care.

A significant investment was made to improve the country’s child health through implementing promotive, preventive, and curative services interventions [33]. Despite the current reduction in childhood mortality observed, the neonatal mortality is stagnant in Ethiopia and SSA [4]. The recent interventions in SSA have slightly improved neonatal mortality from PTB and asphyxia [34]. Investing in expanding NICU services in hospitals, establishing newborn corners in health centres, and expanding community-based newborn care is expected to contribute to the reduction of NMR. However, there are still gaps in providing quality services for neonates, limited competency of health professionals, an absence of neonatal units in some health facilities, and low coverage of skilled delivery. NICU must also be better equipped, and the services must be expanded to health centres [33]. Moreover, coverage of essential interventions, such as case management of acute respiratory illnesses and implementation of KMC services, are low.

The 2017 WHO update recommends proven interventions to decrease sepsis-related deaths, prematurity, and birth asphyxia. These are essential neonatal care immediately after birth, newborn immunisation, newborn resuscitation, management of suspected neonatal sepsis, and preterm/low birth weight, such as prevention of hypothermia, initiation of kangaroo mother care, and thermal maintenance for preterm [35]. Moreover, the quality of ANC care services, early detection, and management of maternal obstetrical complications avert preventable neonatal mortalities [36]. Studies have revealed that more than 80% of the neonatal deaths in SSA could be prevented using the available intervention [34]. Therefore, emphasis should be given to filling the remaining gaps through continuous training of health professionals and improving infrastructure to support preventive and curative services at facility and community levels.

In countries, such as Ethiopia, which lack vital statistics generated from the civil registration system, data from HDSS is a conventional data source for producing timely, continuous, and comprehensive information related to trends of neonatal health problems and their causes. This provides a platform for various health system innovations and interventions. Moreover, most deaths in Ethiopia occur at home; therefore, HDSS data is a reliable method for community diagnoses of significant causes of death.

The limitation of this method is that the collected data depend on the participant who provided this information; therefore, reporting error and recall bias may be introduced. Underreporting of neonatal death is also commonly related to some traditions and beliefs, which may affect the reported rate of neonatal mortality. Causes of death are based on the physician’s agreement and subjective reports from the participants. This can lead to misclassification. Furthermore, we did not undertake regression analyses to identify possible causal factors.

## Conclusion and recommendations

Our analyses show that neonatal mortality is still high, even though it is lower than the national estimates. A fast-declining trend is needed to achieve the SDG in 2030. Sepsis, pre-term birth, and its complications, diseases related to the perinatal period, birth asphyxia, perinatal respiratory disorders, and neonatal pneumonia are the leading causes of neonatal mortality. Deaths within the first seven days of life account for the highest percentage of all neonatal deaths. Facility- and community-based health services should target the promotion of neonatal health and improve their management of neonatal health problems, especially for the leading causes of neonatal deaths. Increasing community awareness to identify early danger signs of the leading causes of neonatal death and referral and improving the healthcare-seeking behaviour of the community is critical to avert neonatal mortality.

## Paper context

This descriptive study reports neonatal mortality data collected from a Health and Demographic Surveillance System site in Ethiopia (2010–2017). The average neonatal mortality rate was 17/1000 live births. There was a 25% increase between 2010 and 2017. The majority of mortality occurred during the first week of life, and most deaths occurred at home. Leading causes of death were infection, pre-term birth, and perinatal disorders. Neonatal mortality requires urgent attention and action in Ethiopia.
